# Evaluating Large Language Models in Interpreting MRI Reports and Recommending Treatment for Vestibular Schwannoma

**DOI:** 10.3390/diagnostics15222841

**Published:** 2025-11-10

**Authors:** Arthur H. A. Sales, Christine Julia Gizaw, Jürgen Beck, Jürgen Grauvogel

**Affiliations:** Department of Neurosurgery, Faculty of Medicine, University of Freiburg, 79106 Freiburg, Germany; christine.gizaw@uniklinik-freiburg.de (C.J.G.); j.beck@uniklinik-freiburg.de (J.B.); juergen.grauvogel@uniklinik-freiburg.de (J.G.)

**Keywords:** large language models, artificial intelligence, vestibular schwannoma, MRI reports analysis, GPT-4, Gemini, Bing

## Abstract

**Background/Objectives**: The use of large language models (LLMs) by patients seeking information about their diagnosis and treatment is rapidly increasing. While their application in healthcare is still under scientific investigation, the demand for these models is expected to grow significantly in the coming years. This study evaluates the accuracy of three publicly available AI tools—GPT-4, Gemini, and Bing—in interpreting MRI reports and suggesting treatments for patients with vestibular schwannomas (VS). To evaluate and compare the diagnostic accuracy and treatment recommendations provided by GPT-4, Gemini, and Bing for patients with VS based on MRI reports, while addressing the growing use of these tools by patients seeking medical information. **Methods**: This retrospective study included 35 consecutive patients with VS treated at a university-based neurosurgery department. Anonymized MRI reports in German were translated to English, and AI tools were prompted with five standardized verbal prompts for diagnoses and treatment recommendations. Diagnostic accuracy, differential diagnoses, and treatment recommendations were assessed and compared. **Results**: Thirty-five patients (mean age, 57 years ± 13; 18 men) were included. GPT-4 achieved the highest diagnostic accuracy for VS at 97.14% (34/35), followed by Gemini at 88.57% (31/35), and Bing at 85.71% (30/35). GPT-4 provided the most accurate treatment recommendations (57.1%, 20/35), compared to Gemini (45.7%, 16/35) and Bing (31.4%, 11/35). GPT-4 correctly recommended surgery in 60% of cases (21/35), compared to 51.4% for Bing (18/35) and 45.7% for Gemini (16/35). The difference between GPT-4 and Bing was statistically significant (*p*-value: 0.02). **Conclusions**: GPT-4 outperformed Gemini and Bing in interpreting MRI reports and providing treatment recommendations for VS. Although the AI tools demonstrated good diagnostic accuracy, their treatment recommendations were less precise than those made by an interdisciplinary tumor board. This study highlights the growing role of AI tools in patient-driven healthcare inquiries.

## 1. Introduction

The use of commercially available AI tools that use natural language processing (NLP) in the context of healthcare has been an object of interest in previous studies [1,2,3,4,5,6,7,8,9,10,11,12,13,14,15,16].

These tools have the advantage of offering an easy-to-use interface that does not require programming language skills, since they understand commands through natural language processing, facilitating their use among physicians and patients.

The fact that these tools utilize an immeasurable amount of online content, such as books, articles, and expert opinions with varying levels of evidence, raises uncertainties about the accuracy of the information generated in response to medical queries and the extent to which these responses can be applied to individual cases. As these tools continue to evolve, their use by patients seeking insights into their diagnosis and treatment is expected to increase substantially in the coming years, highlighting critical concerns regarding the accuracy and dependability of the recommendations they provide, which may positively or negatively influence the doctor-patient relationship.

Vestibular Schwannoma (VS) is a tumor derived from Schwann cells arising from the vestibulocochlear nerve [17,18,19]. This tumor is located in the internal auditory canal and/or cerebellopontine angle and classically manifests with unilateral sensorineural hearing loss [17]. The main diagnostic tool used to investigate patients with suspected VS is the magnetic resonance imaging (MRI) due to its high sensitivity, specificity, and cost-effectiveness [1,20].

The use of AI tools for speech recognition, categorization and extraction of information from radiology reports has been described in the literature [1,2,3,4,5]. This is especially true for AI tools that are based on large language models (LLM), e.g., Generative Pre-trained Transformer version 4 (GPT-4), Google’s Gemini, and GPT-based Microsoft’s Bing [1,2,3,4,5]. In this work, we compared the accuracy of three publicly available LLM (GPT-4, Gemini and Bing) in interpreting MRI reports of patients with VS written in two languages (German and English). In addition, we have evaluated treatment recommendations based only on these MRI findings. To the best of our knowledge, this is the first study that evaluates the role of LLM-based AI tools in both interpreting MRI reports and suggesting treatments for neurosurgical pathologies.

## 2. Materials and Methods

### 2.1. Patients and MRI Report Data Preparation

Thirty-five consecutive patients with vestibular schwannoma who were followed in the skull base clinic of the Department of Neurosurgery at the University Hospital of Freiburg in 2023 were included in this study. The exclusion criteria comprised missing written MRI reports, incomplete radiological documentation, or poor-quality imaging data. Tumor size or stage was not used as an inclusion criterion, resulting in a heterogeneous cohort encompassing both intracanalicular and large cerebellopontine-angle lesions.

**Figure 1:**
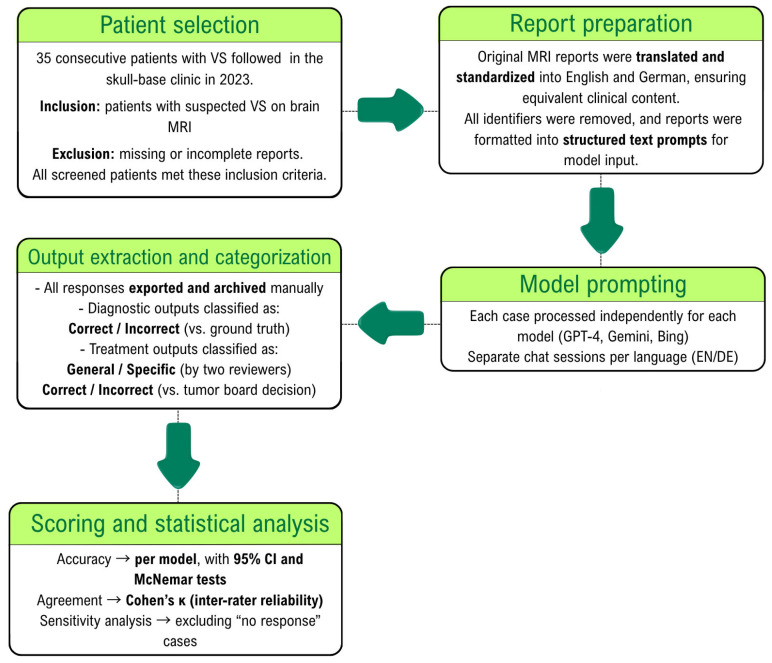
Study workflow from case selection to model evaluation.

All MRI reports were originally written in German and subsequently translated into English through a standardized human-assisted process (Figure 1). Each patient’s report was therefore available in two linguistically parallel forms (35 German and 35 English), allowing for paired analysis across languages. MRI report data was anonymized and converted from Portable Document Format (PDF) to text format. Since the primary outcome of this research is the accuracy of AI tools in diagnosing VS based on MRI reports, the terms ‘acoustic neuroma,’ ‘vestibular schwannoma,’ and any other synonyms directly referring to the etiological diagnosis were replaced by the word ‘lesion’ and its German synonyms in the findings section of the MRI report. Additionally, the conclusion section was excluded from the prompt for the same reason. During this process, no patient identifiers (such as names or addresses) were transferred or manually copied. Two board-certified neurosurgeons independently verified the resulting text files to ensure complete removal of personal data and preservation of the original report content and structure.

### 2.2. Verbal Prompts

We used 5 prompts in English to elicit responses related to diagnosis and treatment, as shown in Table 1.

**Figure 2:**
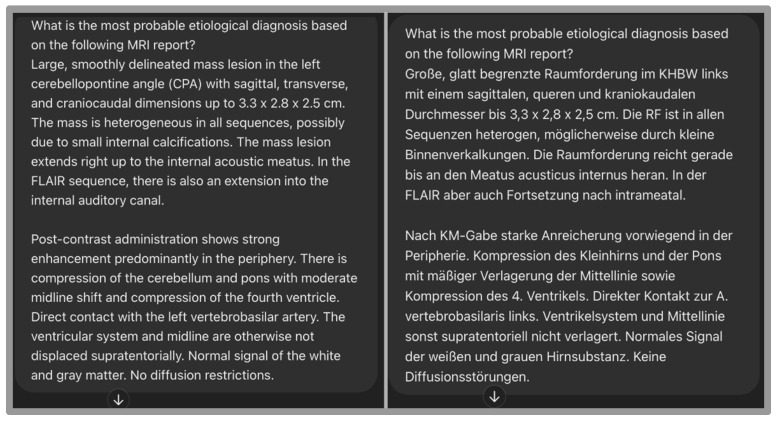
Example of the same prompt with MRI reports written in English and German (original language of the MRI report).

The prompts were used twice for each patient to investigate response changes using the same MRI reports written in two different languages (English and German). We decided to also evaluate the accuracy of the model for interpreting MRI reports in German, as this was the original language used in the reports. See Figure 2. Each case was processed once per language and per model in an independent chat session to prevent memory carry-over effects. To assess output stability, five representative cases were repeated three times for each model under identical conditions. Reproducibility was quantified as the proportion of identical outputs across repetitions.

Prompts 1–4 were zero-shot queries, meaning the models received no prior contextual information other than the MRI report itself. Prompt 5 constituted a prompt-chaining query because it explicitly incorporated the diagnosis revealed in the previous response (‘considering that this is a vestibular schwannoma …’) to elicit a more refined, case-specific treatment recommendation. This design tested the models’ ability to integrate newly provided clinical facts into subsequent reasoning.

### 2.3. Outcomes and Statistical Analysis

The primary outcome was the diagnostic accuracy of each large language model (GPT-4, Gemini, and Bing) in identifying the correct etiological diagnosis of vestibular schwannoma from MRI report text.

Secondary outcomes included:

- The accuracy of AI-generated treatment recommendations relative to the interdisciplinary skull-base tumor board decision;
- The ability of each model to generate specific versus general treatment suggestions (e.g., “microsurgical resection” vs. “treatment required”);
- The proportion of cases in which each model proposed plausible differential diagnoses for cerebellopontine angle lesions;
- The presence of language-related performance differences between German and English report versions.

Analyses were performed using GPT-4 (OpenAI, May 2024 release, accessed via ChatGPT interface in January 2025), Gemini (Google, version 1.5 Pro release June 2024, accessed via Gemini web interface in January 2025) and Bing Copilot (Microsoft GPT-4-based model, accessed January 2025 via web interface). 

All secondary analyses were exploratory and not adjusted for multiplicity. Accuracy values are reported with 95% confidence intervals (Wilson’s method). Pairwise comparisons of model performance and language-related accuracy differences were assessed using McNemar’s test on case-level paired data. A *p*-value < 0.05 was considered significant. Data are presented as mean (standard deviation), median (interquartile range), or number of patients (percentage). Model outputs explicitly declining to provide an etiological diagnosis (‘I cannot make a clinical judgment’) were coded as non-answers and treated as incorrect in the primary analysis. A sensitivity analysis excluding these cases was also performed. All analyses were performed using IBM SPSS Statistics version 27.0 (IBM Corp., Armonk, NY, USA) and R version 4.5.0 (R Core Team, 2025).

### 2.4. Ground Truth

The ground truth for diagnostic accuracy was defined through a two-step expert review. The initial MRI diagnosis was provided by a board-certified neuroradiologist. All MRI scans were independently reassessed by a board-certified neurosurgeon experienced in skull-base imaging, blinded to the neuroradiologist’s interpretation. In the single case of diagnostic disagreement, a third neurosurgeon adjudicated the final label. Because this process represented a hierarchical review rather than parallel ratings, inter-rater agreement was expressed as raw percentage agreement rather than Cohen’s kappa.

The ground truth for overall treatment recommendations was defined as the consensus decision of the interdisciplinary skull-base tumor board at the University of Freiburg, which serves as the institutional gold standard for case management decisions.

For the classification of treatment recommendations as specific versus general, two board-certified neurosurgeons independently assessed each case, with a third reviewer resolving any disagreement. Inter-rater reliability for this categorical classification was assessed using Cohen’s kappa coefficient.

### 2.5. Ethical Issues

This research was conducted in compliance with The Code of Ethics of the World Medical Association (Declaration of Helsinki) for experiments involving human subjects. The local medical ethics committee approved this study (23-1393-S1-retro). Written informed consent was not required due to the retrospective study design.

## 3. Results

All 35 screened patients with vestibular schwannoma had written MRI reports and were included in the analysis. No cases were excluded.

### 3.1. Diagnostic Accuracy for Acoustic Neuroma

Regarding the outputs to prompt 1 (What is the most probable etiological diagnosis based on the following MRI report?) and considering the diagnosis of VS as the ground truth, we obtained the following results shown in Table 2.

**Figure 3:**
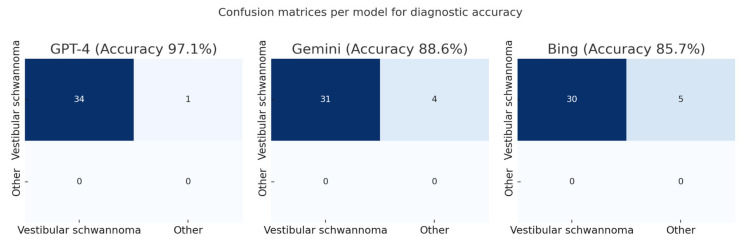
Model-level confusion matrices for diagnostic classification.

**Figure 4:**
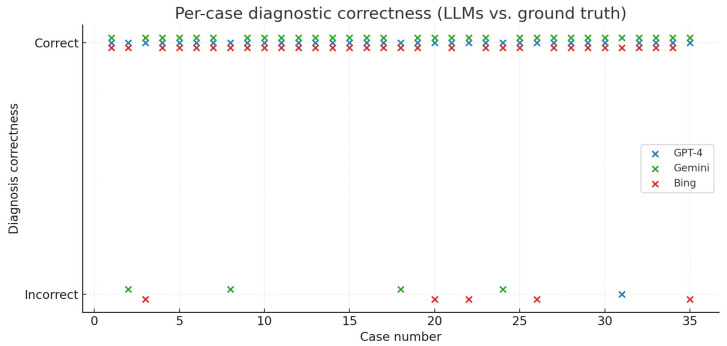
Case-level comparison of diagnostic correctness across models.

GPT-4 correctly identified vestibular schwannoma in 34/35 cases (97.1%), Gemini in 31/35 cases (88.6%), and Bing in 30/35 cases (85.7%) (Figure 3 and Figure 4)

Pairwise McNemar comparisons showed no statistically significant differences:

- GPT-4 vs. Gemini: 4 vs. 1 discordant pairs; odds ratio 4.00 (95% CI 0.45–35.79); *p* = 0.375;
- GPT-4 vs. Bing: 5 vs. 1 discordant pairs; odds ratio 5.00 (95% CI 0.58–42.80); *p* = 0.219;
- Gemini vs. Bing: 5 vs. 4 discordant pairs; odds ratio 1.25 (95% CI 0.34–4.66); *p* = 1.000.

The corresponding absolute differences in accuracy (unpaired) were 8.6% for GPT-4 vs. Gemini and 11.4% for GPT-4 vs. Bing.

When cerebellopontine angle meningioma, the main differential diagnosis of vestibular schwannoma, was additionally accepted as a plausible first diagnosis, accuracy increased to 100% for GPT-4, 97.1% for Gemini, and 88.6% for Bing, without altering the relative ranking of the models.

To assess the reproducibility of diagnostic outputs across models, five representative cases were re-evaluated in multiple runs. Across 45 repeated runs (5 cases × 3 repetitions × 3 models), 44 yielded identical diagnostic outputs (97.8%; 95% CI 88.5–99.9%), corresponding to a variability of about 2% and indicating high reproducibility across models.

GPT-4 identified meningioma as the most probable diagnosis in 1 case, Gemini in 3 cases, and Bing in 1 case. Additionally, Bing provided implausible diagnoses in 3 cases (intracerebral hemorrhage, intradural extramedullary spinal tumor, and intramedullary spinal tumor). GPT-4 and Gemini did not provide any implausible diagnoses. Agreement between the neuroradiologist and neurosurgeon who established the diagnostic ground truth was 34/35 (97.1%; 95% CI 85.5–99.5%), with one case requiring adjudication by a third neurosurgeon.

### 3.2. Differential Diagnoses

With respect to the power of AI tools in suggesting plausible differential diagnoses for the lesion described on MRI reports, we obtained the following results (Table 3):

Meningioma was the second most probable diagnosis for all three AI models. GPT-4 and Bing identified meningioma in 28 cases, while Gemini did so in 30 cases. Bing had the highest rate of implausible diagnoses (3 cases), while GPT-4 and Gemini did not provide implausible diagnoses.

Epidermoid cyst was the third most probable differential diagnosis in all three AI models, followed by cholesteatoma and facial nerve schwannoma (Table 4). We considered “infection” as an implausible diagnosis since it is a very unspecific term that does not directly relate to the described lesions on MRI reports. On the other hand, an abscess would be considered a plausible differential diagnosis because of its mass effect, which can present similarly to other lesions on MRI.

### 3.3. Treatment Recommendations

Prompt 3 inquired the AI tools about treatment recommendations. We classified the outputs answering queries about treatment recommendations into two categories: general recommendations and specific treatment recommendations. General recommendations were based on the supposed diagnosis, providing general treatment guidelines without considering the particular patient whose MRI report was described in the prompt. Specific treatment recommendations included precise suggestions such as surgery, wait and scan, or radiotherapy tailored to treat the particular patient. See Table 5.

Bing was the AI tool with the highest rate of general treatment recommendations (23 cases), followed by Gemini (18 cases). GPT-4 provided general treatment recommendations in only 8 cases. GPT-4 indicated surgery in 26 cases, Gemini in 13 cases and Bing in 11 cases. Inter-rater agreement for the general-vs-specific classification was high across all model outputs: κ = 0.92 (95% CI 0.77–1.00) for GPT-4, κ = 0.94 (95% CI 0.83–1.00) for Gemini, and κ = 0.94 (95% CI 0.82–1.00) for Bing. Only three model-case combinations (one per model; 3/105, 2.9%) required adjudication by the third reviewer.

After revealing the etiological diagnosis (acoustic neuroma) in prompt 5, there was a slight trend towards recommending specific treatment modalities by GPT-4 and Gemini, while Bing increased the rate of general treatment recommendations. See Table 6.

**Figure 5:**
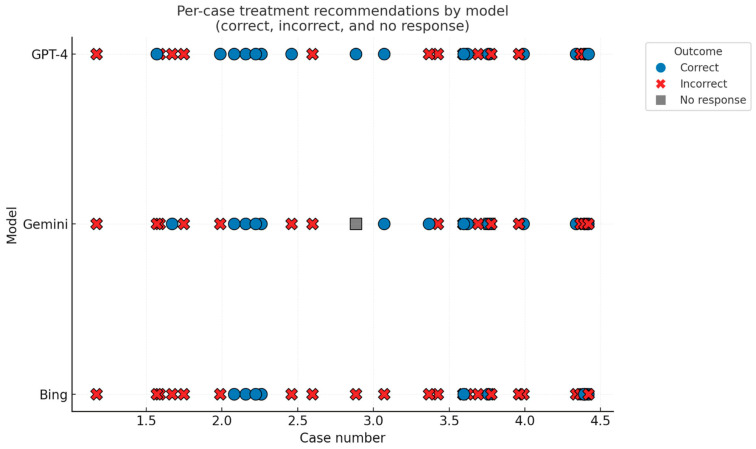
Waterfall plot showing per-case correctness of treatment recommendations.

**Figure 6:**
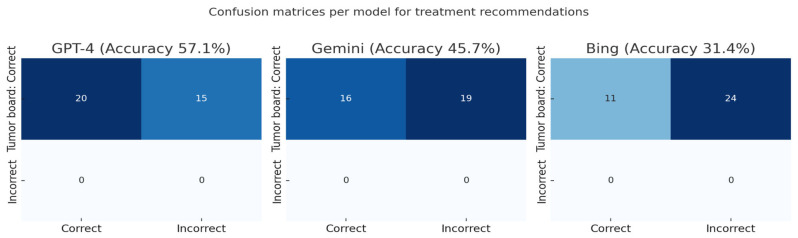
Model-level confusion matrices for treatment recommendations.

Considering the skull-base tumor board recommendations as the ground truth and counting non-responses as incorrect, GPT-4 achieved 20/35 correct treatment recommendations (57.1%, 95% CI 40.9–72.0), Gemini 16/35 (45.7%, 95% CI 30.5–61.8), and Bing 11/35 (31.4%, 95% CI 18.6–48.0). Pairwise McNemar tests showed no significant difference between GPT-4 and Gemini (6 vs. 2 discordant cases; *p* = 0.289; OR 3.0, 95% CI 0.61–14.9) and between Gemini and Bing (7 vs. 2; *p* = 0.180; OR 3.5, 95% CI 0.73–16.9). GPT-4, however, outperformed Bing (11 vs. 2 discordant cases; *p* = 0.022; OR 5.5, 95% CI 1.22–24.8). See Figure 5 and Figure 6.

Excluding the single non-response did not materially affect results: GPT-4 (58.8%, 95% CI 42.3–73.6), Gemini (47.1%, 95% CI 31.9–62.8), and Bing (32.4%, 95% CI 19.1–49.2) maintained the same relative performance ranking. Pairwise McNemar comparisons remained unchanged, confirming that non-responses had no impact on the direction or significance of results.

**Figure 7:**
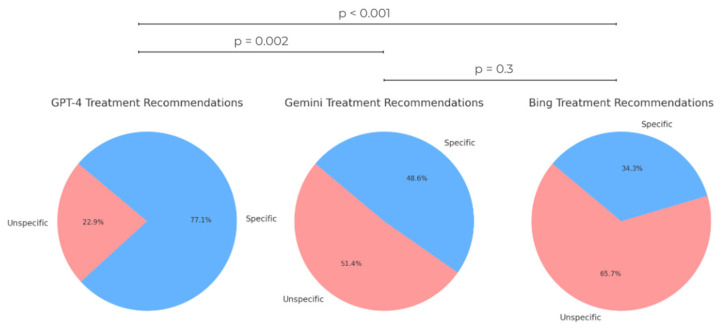
Rates of specific and unspecific treatment recommendations of AI Tools.

The rates of unspecific treatment recommendations (radiotherapy, surgery, or wait and scan), based on treatment guidelines and not considering the individual patient, were also evaluated. GPT-4 recommended specific treatment measures considering the MRI report in 77.1% of cases, Gemini in 48.8% of cases, and Bing in only 34.3% of cases. The difference in rates of specific treatment recommendation between GPT-4 and other AI tools was statistically significant, while the difference between Gemini and Bing did not show statistical significance (Figure 7).

### 3.4. Surgical Indication

Prompt 4 inquired about surgical indication based solely on MRI reports. GPT-4 indicated surgery in 26 cases and did not indicate it in 9 cases. Gemini indicated surgery in 13 cases and did not answer the question in 6 cases, citing insufficient information to make a decision. Bing indicated surgery in 17 cases and did not answer the question in 15 cases.

Considering the treatment recommendations of our interdisciplinary skull base tumor board as the ground truth, GPT-4 presented the highest rate of accurate surgical indication based solely on MRI reports (60%). Gemini indicated surgery properly in 45.7% of cases, while Bing did so in 51.4% of cases. The difference between AI tools was not statistically significant.

Regarding the rates of no response to Prompt 4 (“Would you recommend surgical treatment in this particular case?”), GPT-4 provided responses regarding surgical indication for all patients, whereas Gemini did not answer this question in 17.1% of cases, and Bing failed to respond in 42.9% of cases. The difference in rates of no response regarding surgical treatment between GPT-4 and Bing was statistically significant (*p*-value < 0.001).

### 3.5. Language Issues

The use of MRI reports in the original language (German) did not impact the accuracy of diagnosis by any of the AI tools. For GPT, accuracy was identical in German and English (34/35, 97.1% for both; McNemar *p* = 1.000). Gemini showed a non-significant improvement when prompted in English (from 31/35, 88.6% to 33/35, 94.3%; McNemar *p* = 0.625). Bing also showed a non-significant trend toward higher accuracy in English (from 30/35, 85.7% to 33/35, 94.3%; McNemar *p* = 0.25).

## 4. Discussion

This study addresses an increasingly relevant issue in modern clinical practice: the growing use of LLMs by patients seeking medical advice and bringing these AI-generated recommendations into consultations. Understanding the reliability and limitations of these tools is crucial for physicians, as their outputs must always be interpreted in the context of individualized clinical decision-making.

The results of this study demonstrate that GPT-4 outperforms Gemini and Bing in interpreting MRI reports of patients with VS and providing specific treatment recommendations based solely on these reports. Previous studies have investigated the accuracy of AI models in diagnosing medical conditions [13,21,22,23]. Kanjee et al. investigated the power of GPT-4 in diagnosing 70 challenging medical cases from the New England Journal of Medicine clinicopathologic conferences and reported an accuracy rate of 39% (27/70) of cases. In addition, in 64% of cases (45/70), the model included the final diagnosis in its differential list [24]. Another study compared the accuracy of GPT-4 and GPT-4 with vision (GPT-4V) to that of board-certified radiologists in diagnosing 32 consecutive “Freiburg Neuropathology Case Conference” cases from the journal Clinical Neuroradiology. The study reported that GPT-4 had a lower diagnostic accuracy than each radiologist; however, the difference was not statistically significant [25]. Our study demonstrated that GPT-4 achieved a diagnostic accuracy of 97.14% for VS, higher than Gemini (88.57%) and Bing (85.71%). This higher performance of GPT-4 compared to other publicly available AI models in diagnosing medical conditions was echoed in other studies [2,26,27]. However, it is important to emphasize that all models performed well and the difference in accuracy rates between them was not statistically significant. The present results should be interpreted in the context of the model versions evaluated. Subsequent releases, including GPT-5 and Gemini 2.5, are likely to show different performance characteristics as training data and alignment methods continue to improve.

GPT-4’s higher accuracy likely reflects a larger multimodal training corpus and more refined alignment procedures optimized for complex reasoning tasks. Gemini, although trained on comparable text data, appears less domain-specific in medical language. Bing, which relies on a search-augmented GPT architecture, may incorporate non-curated web content during response generation, occasionally leading to implausible outputs. These architectural and alignment differences may partly explain the observed performance gradient.

Differentiating vestibular schwannoma from cerebellopontine angle meningioma is challenging because both share similar MRI signal patterns and contrast enhancement. The models recognized textual cues such as ‘widening of the internal auditory canal’ or ‘dural tail,’ reflecting radiological reasoning. Their occasional identification of meningioma as the main etiological diagnosis demonstrated realistic diagnostic behavior, as this distinction can be difficult even for expert neuroradiologists.

No patients were excluded from the analysis, as all screened cases had written MRI reports available. The relatively small sample size reflects the exploratory design of this proof-of-concept study, which aimed primarily to assess whether publicly available large language models could accurately interpret standardized MRI reports and to compare their relative diagnostic performance. While all evaluated LLMs demonstrated high diagnostic accuracy, their susceptibility to data-source bias and misinterpretation remains an ethical concern. Since LLMs generate probabilistic text without transparent reasoning or certified medical validation, their use in clinical workflows must always involve human oversight. Transparent auditing of model outputs and continuous ethical monitoring are essential to prevent potential harm from over-reliance on AI-generated medical advice.

In the language comparison analysis, diagnostic accuracy across all three models remained largely consistent between German and English prompts. These results indicate that the prompt language did not materially affect diagnostic performance, although English prompts occasionally rescued misclassifications observed with German input. This finding is significant as it underscores the robustness of these AI models in processing medical information across different languages, thus broadening their applicability in diverse clinical settings. This result was also confirmed by a study that demonstrated the efficiency of AI tools in assigning BI-RADS categories across reports written in three different languages [2].

Mohammad-Rahimi et al. [26] assessed the responses of six different artificial intelligence (AI) chatbots (Bing, GPT-3.5, GPT-4, Google Bard, Claude, Sage) to controversial and difficult questions in oral pathology, oral medicine, and oral radiology. They found that GPT-4 was the most superior chatbot in providing high-quality information on controversial topics across various dental disciplines [26]. In our study, implausible diagnoses such as intramedullary spinal tumor or cerebral microangiopathy in cerebellopontine-angle reports were classified as hallucinations. These errors likely reflect limited domain representation and overgeneralization from unrelated medical contexts. 

One of the main practical applications of this study is the simulation of the responses that patients would obtain if they uploaded their MRI report. The use of the internet as a source of information for medical conditions has become a common practice among patients, and in the era of large language models, this usage is expected to increase significantly, as well as patients’ trust in the information that has been extracted. In this study, we used prompts written in scientific language to extract the most accurate responses and evaluate the LLMs at their full potential. For healthcare providers, it will become increasingly important to understand the outputs of such LLMs for various medical conditions, as patients are likely to use these tools more frequently in the future. This familiarity is essential for clinicians to navigate consultations effectively, recognizing the (realistic or unrealistic) expectations and preconceptions that patients may bring to the encounter, potentially influenced by AI-generated advice. Future studies reporting the frequency of medical queries directed at LLMs could provide further insights into this growing trend.

Regarding treatment recommendations, GPT-4 provided the most accurate recommendations at 57.1%, followed by Gemini at 45.7% and Bing at 31.4%. As mentioned in the methods section, we considered the recommendations of our interdisciplinary skull base tumor board, composed of several board-certified physicians, as the ground truth. This approach is supported by the fact that the management of vestibular schwannomas follows a relatively well-defined decision-making pathway, with three main treatment options (surgery, radiotherapy, wait and scan). Key factors influencing these decisions include compression of the brain stem, tumor size, patient age, and specific symptoms, ensuring a structured and consistent framework for evaluating cases. The difference in accuracy rates of treatment recommendation between GPT-4 and Bing was statistically significant (*p*-value: 0.02). It is important to mention that even though Bing is based on GPT models, they can exhibit significant performance differences due to various factors. GPT-4 is specifically designed and fine-tuned for a wide range of applications, while Bing is tailored more towards general search and information retrieval tasks. This specialization in training can lead to substantial differences in performance, particularly in complex and nuanced fields such as medical diagnostics. Moreover, GPT-4 was able to suggest specific treatment measures tailored to individual patients in 77.1% of cases, compared to Gemini (48.6%) and Bing (34.3%). This specificity in treatment recommendations was statistically different from Gemini and Bing, and aligns with findings from previous research [6]. This may reflect differences in training scale, reinforcement alignment, or contextual reasoning capacity. Although these factors could contribute to a more coherent synthesis of clinical cues, such as recognizing terms like brainstem compression or a large cerebellopontine-angle component as indicators of therapeutic urgency, these mechanisms cannot be empirically verified. From a clinical standpoint, this pattern suggests that GPT-4 may approximate multidisciplinary reasoning more closely than the other models and could assist in pre-board case summarization or guideline cross-checking. Nevertheless, its outputs remain non-deterministic and require expert validation before any clinical use. With respect to surgical treatment recommendations based on MRI reports, the examined AI tools performed similarly, with GPT showing an accuracy rate of 60%, compared to 51.4% for Bing and 45.7% for Gemini. Regarding the recommendation of surgical treatment, Bing and Gemini failed to provide responses in some cases (Bing in 42.9% of cases and Gemini in 17.1% of cases). The typical response was: “I am not capable of suggesting medical treatment.” This behavior appeared to occur inconsistently, as the same prompt sometimes elicited this disclaimer, while at other times a treatment recommendation was provided. A possible hypothesis for this phenomenon is that the variability in responses could stem from the limitations in their training data. This study exclusively assessed three publicly accessible closed-source LLMs to mirror real-world patient usage. Future investigations should include open-source and medically fine-tuned models such as LLaMa, BioGPT, and MedPaLM to compare domain-specific performance, interpretability, and auditability [28,29,30]. Such comprehensive benchmarking would elucidate the relative advantages and safety profiles of open versus commercial LLM architectures in neurosurgical contexts.

When faced with complex or ambiguous scenarios that exceed their confidence level, the models might default to a safety mechanism by refusing to provide a recommendation to avoid generating potentially harmful or inaccurate advice. The most immediate clinical applications of LLM-based tools are expected in structured report generation, automated translation of radiological narratives, and standardized extraction of tumor characteristics from free-text MRI reports. This may streamline communication between neuroradiologists and neurosurgeons and accelerate multidisciplinary decision-making. However, these systems also shift the clinician’s workload toward higher-level oversight, validation, and error detection. Rather than replacing expert input, they redefine it by emphasizing supervision, interpretive judgment, and the ethical responsibility to ensure accuracy and patient safety. Moreover, future research should extend the evaluation beyond free-format zero-shot prompts to include structured and role-specific prompting strategies. Examples include prompts enriched with relevant background information (‘Act as a neurosurgeon interpreting this MRI report’) or chain-of-thought scaffolding. Such designs would simulate realistic communication scenarios and enable a more comprehensive assessment of LLM usability in clinical practice.

One limitation of this study is considering the decision of one interdisciplinary tumor board as the ground truth, given that the evaluation of the same patient may lead to different recommendations across different tumor boards. However, we believe that for scientific purposes, using a tumor board from a university hospital composed of multiple board-certified physicians as the ground truth is deemed appropriate. Another limitation of this study is that treatment recommendations were provided based solely on MRI reports, whereas tumor board decisions also take into account other significant factors such as age, the Karnofsky Performance Index, patient symptoms, and neurological deficits. However, this lack of additional information also serves the purpose of simulating patient inquiries when they have only their MRI report and may not consider the aforementioned factors relevant for diagnostic and treatment purposes. Future research should focus on incorporating additional clinical variables to further enhance the performance and applicability of AI in medical diagnostics and treatment planning. In addition, this study was based on a consecutive single-center cohort of 35 patients, which ensures internal consistency but restricts external generalizability. Patient demographics, report style, and institutional wording may differ elsewhere. Multi-center datasets and larger sample sizes are required to confirm reproducibility of accuracy metrics across healthcare systems. LLMs lack intrinsic mechanisms to assess the scientific validity or hierarchy of evidence within their training data. Consequently, they may blend peer-reviewed medical knowledge with unverified online content, producing statements that appear coherent but may be clinically inaccurate. When such generalized information is applied to individual cases, it can result in misleading reassurance or unwarranted concern. This risk is particularly relevant for patients who use publicly available models to interpret their own imaging reports or symptoms. Without professional guidance, they may over- or misinterpret AI-generated advice, underscoring the need for transparent disclaimers and continuous public education about the limitations of these tools.

## 5. Conclusions

This study provides solid evidence that publicly available AI tools (GPT-4, Gemini and Bing) perform well when interpreting MRI reports of patients with VS. With respect to treatment recommendations, GPT-4 performed significantly better than Gemini and Bing, showing greater accuracy and specificity. On the other hand, the occasional generation of implausible diagnoses by Gemini and Bing suggests a need for ongoing refinement of these models to ensure their clinical reliability. This study provides evidence to support communication with patients regarding the current limitations of using LLM in diagnosing and treating neurosurgical pathologies. Future work should focus on expanding the dataset to multi-institutional cohorts, integrating additional clinical variables such as hearing function and performance scores, and exploring multimodal LLM architectures capable of processing both text and imaging data.

## Figures and Tables

**Table 1:** Verbal Prompts.

What is the most probable etiological diagnosis based on the following MRI report?
2. What are the three most probable differential diagnoses for the lesion described in item 1?
3. What is the best indicated treatment for this lesion considering its morphology, location, and size?
4. Would you recommend surgical treatment in this particular case?
5. Considering that this is a vestibular schwannoma, what is the best treatment option for this particular case considering the morphology and location of the lesion described in item 1?

**Table 2:** AI Tools and most probable diagnosis based on MRI report.

	Diagnosis	Frequency (%)
GPT-4	Vestibular Schwannoma	34 (97.14%)
	Meningioma	1 (2.85%)
Gemini	Acoustic Neuroma	31 (88.57%)
	Meningioma	3 (8.57%)
	Pilocytic Astrocytoma	1 (2.85%)
Bing	Vestibular Schwannoma	30 (85.71%)
	Meningioma	1 (2.85%)
	Intracerebral Hemorrhage	1 (2.85%)
	Intradural Extramedullary Spinal Tumor	1 (2.85%)
	Intramedullary Spinal Tumor	1 (2.85%)
	Epidermoid Cyst	1 (2.85%)

**Table 3:** Second most probable differential diagnosis suggested by AI tools based on MRI reports.

Diagnosis	GPT	Gemini	Bing
Vestibular Schwannoma	1	3	1
Arachnoid Cyst	0	0	1
Cerebral Microangiopathy *	0	0	1
Cholesteatoma	0	0	1
Dumbbell Schwannoma	0	0	1
Epidermoid Cyst	1	0	0
Facial Nerve Schwannoma	5	0	0
Intradural Extramedullary Spinal Tumor *	0	0	1
Meniere Disease *	0	0	1
Meningioma	28	30	28
Vascular Malformation	0	2	0

* Implausible diagnoses based on MRI reports.

**Table 4:** Third most probable differential diagnosis suggested by AI tools based on MRI reports.

Diagnosis	GPT	Gemini	Bing
Vestibular Schwannoma	0	2	1
Aneurysm	0	0	4
Cavernoma	0	0	1
Cerebral Amyloid Angiopathy *	0	0	1
Cholesteatoma	1	16	2
Chondroma	0	2	0
Epidermoid Cyst	14	6	14
Exostosis of the IAC	0	0	1
Facial Nerve Schwannoma	11	3	2
Brainstem glioma	0	0	1
Glomus Tumor	0	2	2
Hemangioblastoma	0	0	2
Infection *	0	2	1
Meningioma	6	0	1
Metastasis	2	0	2
Trigeminal Schwannoma	1	0	0
Vascular Malformation	0	2	0

* Implausible diagnoses based on MRI reports.

**Table 5:** Treatment recommendations of AI tools based on MRI reports.

Treatment	GPT	Gemini	Bing
Surgery	26	13	11
Radiosurgery	5	2	2
Wait and Scan	1	2	1
General Treatment	8	18	23

**Table 6:** Treatment recommendations of AI tools based on MRI reports after revealing the etiological diagnosis (AN).

Treatment	GPT	Gemini	Bing
Surgery	27	13	10
Radiosurgery	7	3	2
Wait and Scan	0	4	1
General Treatment	7	16	24

## Data Availability

The original contributions presented in this study are included in the article. Further inquiries can be directed to the corresponding author.

## Abbreviations

AI: Artificial Intelligence; MRI: Magnetic Resonance Imaging; VS: Vestibular Schwannoma; GPT-4: Generative Pre-trained Transformer 4; NPL: Natural Language Processing; LLM: Large Language Model.
